# Editorial: Advances in molecular biology knowledge of rectal cancer and forthcoming role of liquid biopsy

**DOI:** 10.3389/fonc.2024.1476174

**Published:** 2024-08-29

**Authors:** Francesca Negri, Letizia Gnetti, Carlo Aschele

**Affiliations:** ^1^ Gastroenterology and Endoscopy Unit, University Hospital of Parma, Parma, Italy; ^2^ Department of Pathology, University Hospital of Parma, Parma, Italy; ^3^ Medical Oncology Unit, Department of Oncology, Ospedale Sant’Andrea, La Spezia, Italy

**Keywords:** rectal cancer, molecular biology, liquid biopsy, prognosis, biomarker

Advances in molecular biology may significantly impact our understanding of rectal cancer and inform the development and positioning of novel therapies (1). Total Neoadjuvant Treatment (TNT) for locally advanced rectal cancer is gaining consensus and has evolved, emphasizing the need of a multidisciplinary strategy. TNT has increased complete response rates, disease-free survival, control of distant metastases and has become a new standard of care (2, 3). Also, a raising amount of reports indicates that a non-operative management (NOM), entailing close surveillance of patients with clinical complete response (cCR) after neoadjuvant therapy, could be an appropriate alternative option to rectal surgery (4). Recently, the International Watch & Wait Database (IWWD) reported superiority in terms of quality of life and a small risk of local inoperable disease recurrence without compromising survival in patients with rectal cancer managed nonoperatively after achieving a cCR following neoadjuvant therapy (5).

Selecting predictive molecular markers is thus becoming even more crucial. The tumor microenvironment plays a critical role in colorectal tumor development, progression and immune escape (6). Stromal cells (i.e. adipocytes, vasculature, lymphocytes) interact with cancer cells and may affect therapy response. As reported in the review by Mirza et al., tumor microenvironment evaluation during treatment may inform on new therapies, uncover responses and tumor resistance. The intratumoral immune contexture is a main factor of clinical outcome in both early- and advanced-stage colorectal cancer (7, 8). Specifically, in rectal cancer a local hot immune signature in the tumor before neoadjuvant therapy is correlated with improved response and prolonged disease-free survival (9). Besides, the 100% complete clinical response rate after programmed cell death protein-1 (PD-1) blockade soundly suggests that the *in situ* innate immune response released and enhanced by immune checkpoint inhibitors treatment can fully eradicate cancer cells precluding recurrences (10). Tumor immune infiltrate has also been described as an independent prognostic marker in a large international cohort of rectal cancer patients with cCR managed nonoperatively and could pave the way for prospective therapeutic trials guided by immunoscore to adapt follow up and/or therapy of NOM patients (11).

Colorectal cancers show genetic variations and clonal evolution, which proffer noteworthy difficulties in selecting appropriate therapies (12). In the traditional approach, the identification and choice of therapy have mainly depended on the employment of invasive tissue biopsies and imaging assays. Currently, core tumor biopsy specimens represent the gold standard biological tissue to identify and analyze predictive biomarkers. However, anatomical feasibility, tumor heterogeneity and cancer progression are major limitations of this single-snapshot approach. Liquid biopsy is increasingly gaining attention as a complementary and potentially alternative non-invasive tool to bypass these limitations.

Liquid biopsy assessment of circulating tumor DNA (ctDNA) is useful for risk stratification and detection of minimal residual disease (MRD) in early colorectal cancer (13). ctDNA can also outline the tumor mutational profile, detect mutations not identified in the tissue biopsies and offer a comprehensive and dynamic evaluation of tumor genetics, classify specific therapeutic targets thus allowing clinicians to monitor disease progression and the efficacy of treatments (13). The introduction of liquid biopsies has endorsed a noteworthy move towards precision medicine in colorectal cancer; the presence of ctDNA in high-risk stage II (T4) and stage III colorectal cancer patients correlates with adverse prognosis both post-surgery and post-adjuvant treatment independent of other conventional clinical-pathological risk factors (13). More recently, Tie and co-authors demonstrated that patients with liver-only metastases undergoing surgical resection had a lower relapse-free survival in the case of ctDNA positivity (14), thus confirming the potential role of serial ctDNA analysis as an immediate marker of therapy activity. As reported by Choi et al., liquid biopsies using extracellular vesicle DNA (evDNA) secreted by tumor cells may be a different source for the detection of cancer driver mutations and a complementary tool for the diagnosis and surveillance of colon cancer patients. Their results showed that evDNA isolated from the plasma of colon cancer patients harboring KRAS G12D and G13D mutations was significantly associated with both CEA level and survival. Unlike fragmented pieces of ‘cell-free’ DNA (cfDNA) shed from apoptotic or necrotic cells, extracellular vesicles arise from viable tumor cells. Therefore, evDNA might reveal the underlying biology of living cancer cells (15, 16) and reflect cancer driver mutations even in the early stages of cancer development (17).

In rectal cancer liquid biopsy could be important in several steps: at the time of diagnosis, for the evaluation of MRD, treatment response and possible acquired resistance and also to modulate treatment during TNT (18). The up-to-date trimodality approach for locally advanced rectal cancer comprises chemotherapy, radiotherapy and surgery and may cause considerable morbidities; moreover, it might not be mandatory for some patients, and fails to prevent disease relapses in others. The main drawback in the present managing of rectal cancer is the absence of consistent and accurate techniques of predicting responsiveness to neoadjuvant therapies without surgical resection and subsequent pathological evaluations. For instance, among patients candidate to sphinter-sparing surgery who demonstrate adequate clinical responses to induction systemic chemotherapy, omitting radiotherapy and its associated toxicities might be a valuable therapeutic option (19). Compared to standard pathological evaluation criteria, ctDNA or modifications in ctDNA could be useful in directing therapeutic decision in this setting of patients. Additionally, a tool to improve the degree of concordance between clinical and pathological complete response could assist a NOM strategy.

In conclusion, the knowledge of tumor microenvironment and immune changes together with introduction of liquid biopsies could offer a measure of these dynamic interfaces, thus enabling the development of immunotherapies and tailored therapies. The inclusion of liquid biopsies in the design of clinical trials, in addition to other analytical modalities such as conventional tissue biopsies, is a crucial component of this development. The Research Topic gives an overview of liquid biopsy and other new technologies and methods as well as emphasizing the clinical usefulness of liquid biopsy, particularly investigating its implication as an analytic, predictive, or MRD marker.
